# What might influence the elderly willingness to participate in “shared elderly care”? A mixed methods study

**DOI:** 10.3389/fmed.2025.1615192

**Published:** 2025-11-18

**Authors:** Hejia Wan, Zilin Zhao, Xinghui Li, Tianyue Xiang, Yifan Qi, Jing Zhang, Yuanmei Qin

**Affiliations:** 1 School of Nursing (Nursing School of Smart Healthcare Industry), Henan University of Chinese Medicine, Zhengzhou, China; 2 School of Information Management, Zhengzhou University, Zhengzhou, China; 3 School of Acupuncture and Massage, Henan University of Chinese Medicine, Zhengzhou, China

**Keywords:** shared elderly care, e-health literacy, machine learning, age-friendly technology, resource allocation, aging society

## Abstract

**Purpose:**

This study aimed to explore the core factors influencing participation in the “shared elderly care” model among urban Chinese seniors and propose targeted solutions to address the challenges of an aging society.

**Methods:**

A mixed-methods study was conducted. A questionnaire survey was conducted among 533 seniors in Zhengzhou. Data on demographic characteristics, health literacy, and environmental factors were analyzed using four machine learning algorithms: logistic regression, random forest, K-nearest neighbor, and support vector machine. Approximately 3 years later, qualitative validation was conducted through six focus group interviews. Themes were extracted using Colaizzi phenomenological analysis, and the predictions were validated.

**Results:**

Five hundred valid questionnaires were collected. The machine learning algorithm results showed that the random forest model had the best predictive performance (AUC = 0.652), revealing that e-health literacy and policy awareness were key drivers (jointly explaining 24.1% of the variance in participation intention), with age, environmental sensitivity, and social influence as significant cofactors. Qualitative analysis confirmed that technology usability and a sense of social belonging were core experiential elements of deep participation.

**Conclusion:**

Addressing the primary obstacles of digital literacy gaps and limited technological accessibility, we propose three countermeasures: increasing publicity and promotion of shared elderly care models; conducting community digital health literacy training; and increasing resource allocation to precisely match needs, thus providing an implementation path for building an inclusive shared elderly care ecosystem.

## Graphical abstract

**Figure:**
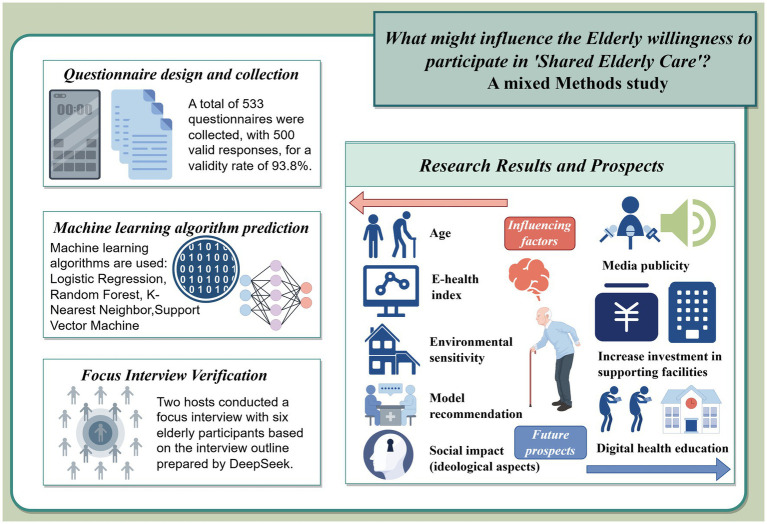
Graphical abstract

## 1 Introduction

In recent years, population aging has become a major global public health issue. As people age, their physical functions gradually decline, leading to a wide variety of diseases with long disease courses, which in turn affect their daily lives and social functions (1, 2). How to improve the quality of life and social value of the elderly in practice has become a new challenge.

Healthy aging is an important measure to actively respond to the challenges of aging and to mitigate and offset the negative impact of an aging society (3, 4). In addition, a sound and complete elderly care service system is the foundation for building a healthy aging society. In 2019, the World Health Organization (WHO) released the draft of the “Decade of Action for Healthy Aging 2020–2030”, which provides a reference for the construction of a healthy aging society (5). China’s “National Medium- and Long-Term Plan for Actively Responding to Population Aging” proposes to establish and improve a comprehensive and continuous elderly health service system covering health education, preventive health care, disease diagnosis and treatment, rehabilitation care, long-term care and palliative care to promote the physical and mental health of the elderly (6).

Exploring elderly care models under the background of “healthy aging” is a hot issue that needs to be urgently addressed by the Chinese government and the world. At present, “family-based elderly care” has become the mainstream elderly care model in China (7). However, in recent years, many small and nuclear family structures have gradually formed, resulting in the weakening of family-based elderly care functions and the increase of family-based elderly care burdens (8). Therefore, exploring a new home-based elderly care model is crucial for integrating and optimizing social resources for the elderly, achieving precise elderly care services, and improving the quality of life of the elderly. America has implemented “time bank” services. These services have significantly promoted the development of elderly care services through professional training of volunteers and the establishment of a comprehensive management system and relevant rules and regulations (9). In China, medical institutions have adopted the “nurse sharing” method to effectively alleviate the shortage of nursing talents (10).

**Figure 1:**
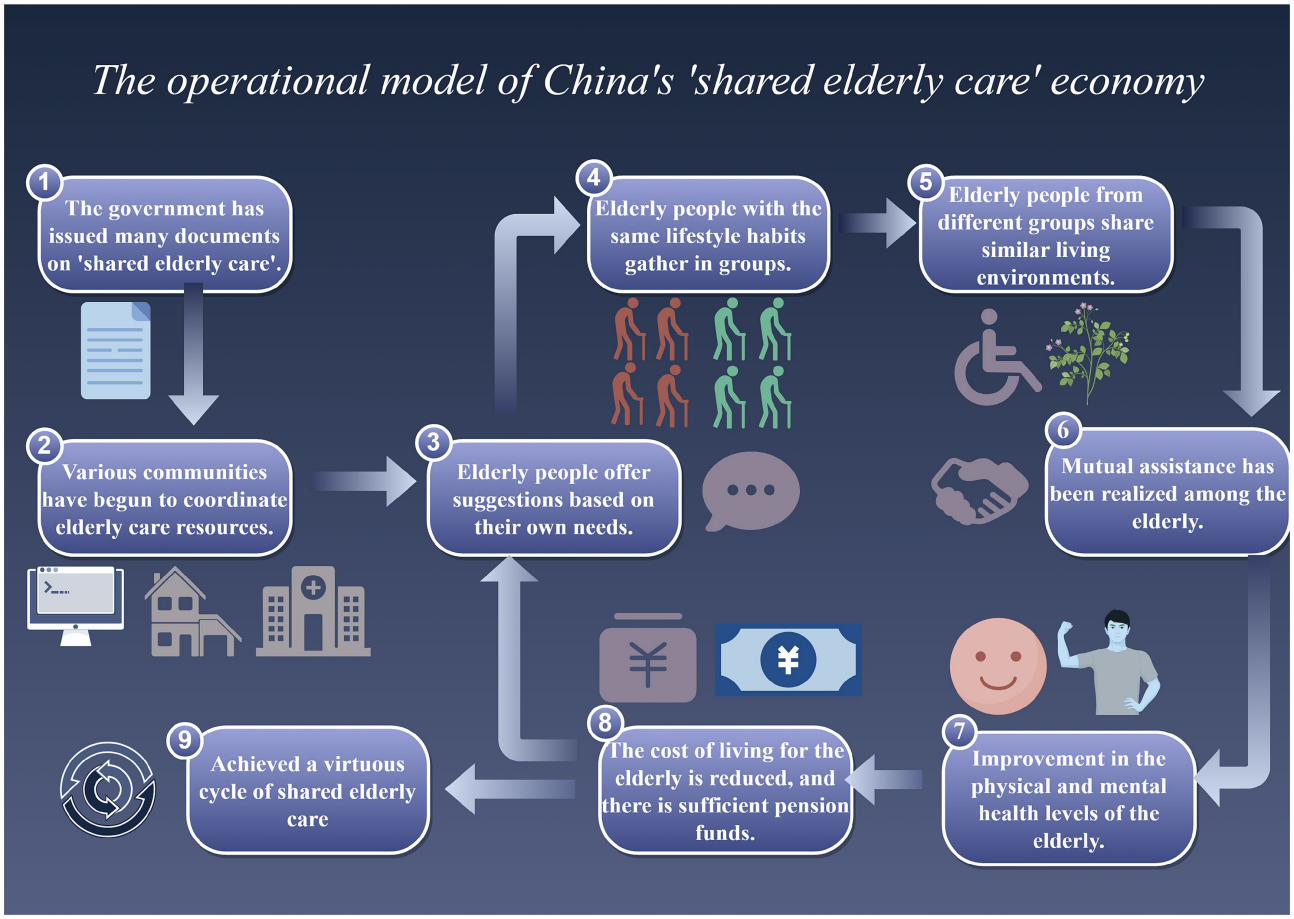
The operational model of China’s shared elderly care economy.

With the increasing popularity of the Internet, the “shared elderly care” model has emerged in China. The characteristics of this model are reflected in two aspects: one is ‘sharing’ and the other is “home-based”. Specifically, the community organizes surplus labor and caring volunteers who are willing to engage in housekeeping services and elderly care services to establish professional service agencies, and allocates and shares resources according to the actual needs of the elderly in the community, realizing an organic combination of supply and demand (11). “Shared elderly care” is based on the theory of sharing economy and the Internet platform. It combines the advantages of home-based elderly care and institutional elderly care, can fully tap the potential of elderly care services, effectively integrate and dispatch the scattered, dispersed and potential elderly care service resources in the community, and realize the integration of “menu-based services” and “precision elderly care” (12), providing necessary services for the elderly in a more effective and convenient way, as shown in Figure 1.

**Figure 2:**
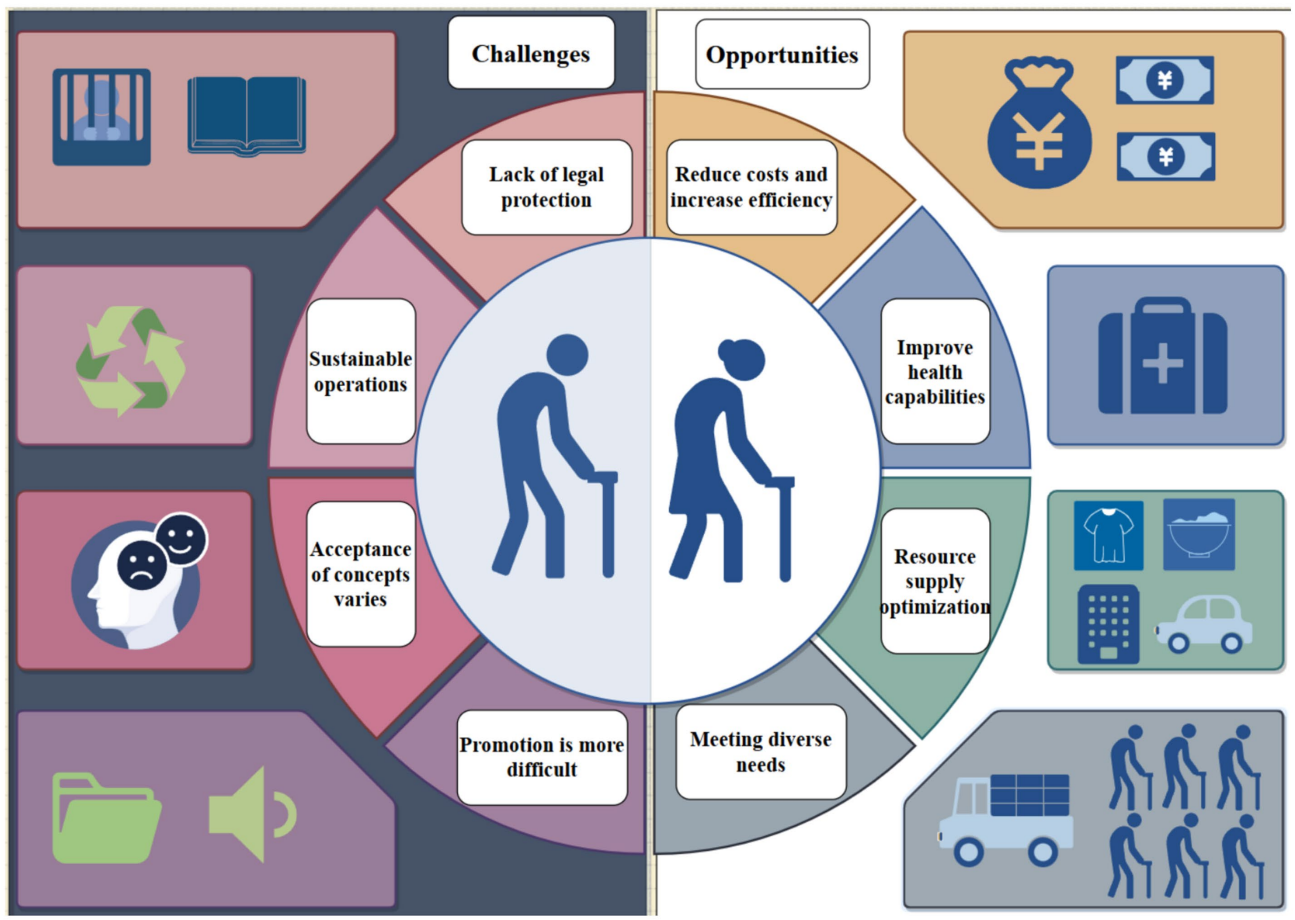
The opportunities and challenges of the shared elderly care model.

At present, China’s “shared elderly care” model is still in the exploratory stage. Some cities have begun to conduct preliminary exploration of this model, providing timely and comprehensive care for the elderly through the “shared home elderly care service model” and “neighborhood sharing” services. These initiatives provide the elderly with faster and more comprehensive home elderly care services, which to a certain extent meet their needs in old age (13). However, since the “shared elderly care” model relies on the Internet, factors such as the limited learning ability and low e-health literacy of the elderly may affect the promotion of shared elderly care to a certain extent. Although there are significant opportunities in the implementation of shared elderly care, there are also considerable limitations (as shown in Figure 2). Therefore, it is crucial to understand the level of willingness of the elderly to ‘shared elderly care’ and the related influencing factors, which will help promote the comprehensive development of the ‘shared elderly care’ model.

Most of the research on ‘shared elderly care’ in China is based on cognitive surveys of practitioners (14). Scholars have used descriptive statistical analysis and logistic regression methods to study willingness. However, the logistic regression method requires only significant factors to be included, while the willingness to participate in shared elderly care is affected by multiple factors, and the combined influence of many non-significant factors may have a greater impact on participation willingness. Therefore, the logistic regression method may not be able to accurately predict and identify the willingness to participate in shared elderly care. In contrast, machine learning methods are better at processing high-dimensional and inseparable data, can model multivariate problems, and are widely used in the discrimination and prediction of categorical data. Commonly used classification models include k-Nearest Neighbor Classification, support vector machines, and random forests. At the same time, the traditional machine learning model logistic regression also performs well in nonlinear models.

Based on this, this study intends to use four machine learning algorithms to model and predict the willingness of urban seniors to participate in shared elderly care. The model’s predictions will be evaluated through internal validation (algorithm comparison) and external validation (focused interviews). Given that this type of research involves human behavioral patterns, and based on a review of relevant domestic and international research literature, we decided to construct a research framework based on the Anderson Behavioral Model. This model can be used to analyze the factors influencing seniors’ willingness to participate in shared elderly care.

## 2 Materials and methods

This study was a hybrid approach. First, data collection and analysis were conducted to compare the strengths of various models. The “distribution of important features” ranked by the best machine learning algorithm was used as the model’s predicted outcome. To validate this outcome, the study used *Deepseek* to develop an interview outline based on the first five indicators and conducted local focused interviews. Two moderators conducted unstructured interviews with six highly educated older adults. The interviews were then summarized using the *Colaizzi* seven-step method, and the analysis results were verified and validated by two experts to ensure accuracy.

**Figure 3:**
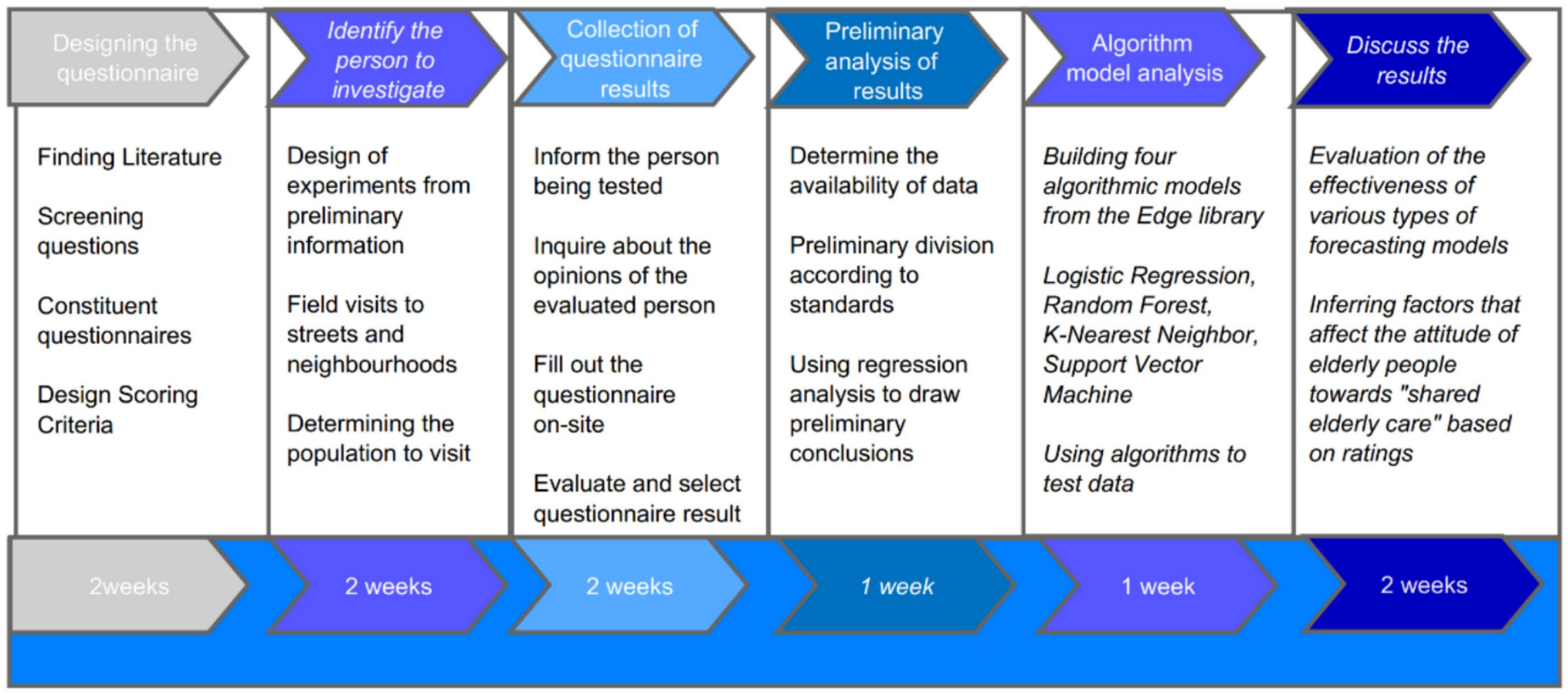
Research progress chart.

The data collection and analysis process for this study consisted of six steps: questionnaire design, survey participant selection, questionnaire result collection, preliminary compilation, results analysis, algorithm model analysis, and discussion of the results. Each stage took 1–2 weeks (Figure 3).

**Figure 4:**
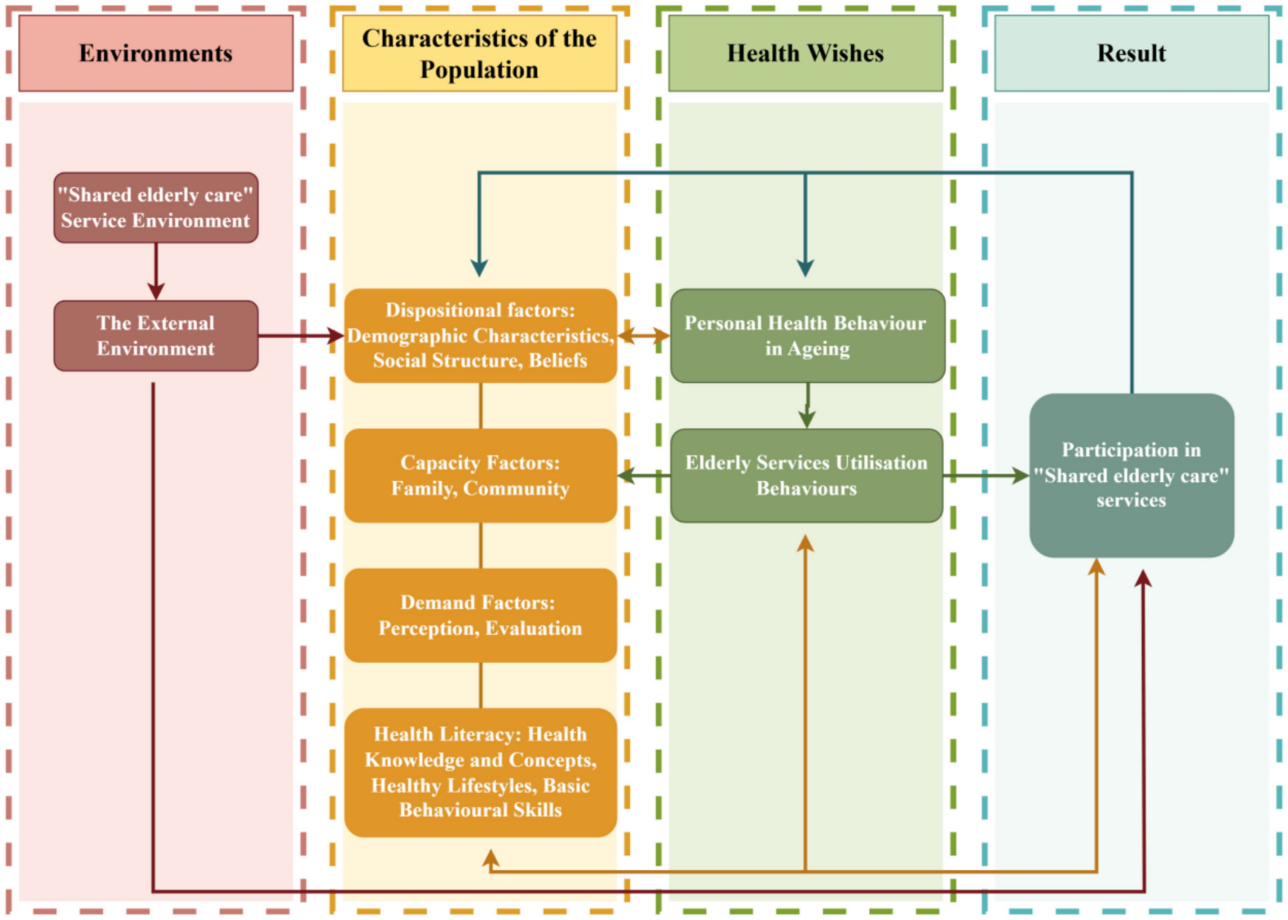
Anderson’s health behavior model-based research framework diagram.

This study uses the Anderson Health Behavior Model as a reference and designs a theoretical framework based on factors such as the environment, population characteristics, health intentions, and outcomes (15). The “Shared Elderly” environment refers to the management environment constructed by current national policies and medical hardware facilities, as well as other external environments. The population characteristics are analyzed from the perspectives of demographic characteristics, ability factors, demand factors, and health literacy (Figure 4).

### 2.1 Study participants

From September 2021 to January 2022, this research team used convenience sampling to select 500 urban elderly residents from *Zhongyuan*, *Huiji*, *Erqi*, *Guancheng*, and *Jinshui* districts of Zhengzhou City to complete a questionnaire survey on their willingness to participate in “share elderly care.”

### 2.2 Inclusion and exclusion criteria

#### 2.2.1 Inclusion criteria

① Living in the community for at least 6 months;
② Age ≥ 60 years;
③ No cognitive or language impairments;
④ Ability and willingness to participate in this study.

#### 2.2.2 Exclusion criteria

- ① Brain metastasis;
- ② History of mental illness;
- ③ History of drug dependence.

### 2.3 Measurement methods

#### 2.3.1 General information questionnaire

Includes age, gender, education level, marital status, living status, physical condition, and health status.

#### 2.3.2 Daily living ability scale (ADL)

This questionnaire was developed by Lawton and Brody in 1969 and is mainly used to assess the subjects' daily living ability. It has a total of 14 items, including the physical self-care scale (six items) and the instrumental daily living ability scale (eight items). A score of less than 16 points is considered completely normal. The higher the score, the worse the self-care ability. The highest score is 64 points. The reliability is good.

#### 2.3.3 Questionnaire on the willingness of the elderly to participate in “shared elderly care”

This questionnaire was compiled by Venkatesh et al. (16) in 2012. Yi et al. (2) and other scholars used the above questionnaire to form the ‘Questionnaire on the Participation of the Elderly in ‘shared elderly care’ and constructed a questionnaire on the willingness of the elderly to participate in “shared aging”. It has a total of 21 items, including seven dimensions: perceived environmental integration, perceived ease of use of technology, perceived cost savings, perceived social impact, perceived usefulness, perceived willingness to participate, and willingness to recommend. The questionnaire uses a 7-point Likert scale. The higher the score, the higher the willingness to participate. The reliability is good.

#### 2.3.4 Electronic health literacy scale (EHLS)

The EHLS was developed by Norman et al. (17) and adapted into Chinese by Guo et al. (18). It is used to assess an individual’s ability to search for, understand, evaluate, and use online health information. The scale consists of 8 items, each scored from 1 to 5 points, with a score of >32 considered acceptable (19). Higher scores indicate higher electronic health literacy. The internal consistency of the scale, with a Cronbach’s *α* coefficient of 0.913, indicates good reliability and validity.

### 2.4 Data collection

Methods: Survey personnel received unified training prior to the survey. Survey personnel distributed questionnaires electronically (*Wen Juan Xing*: The most commonly used questionnaire collection and analysis software in China) or *in person* using standardized instructions. The study objectives and procedures were explained to the elderly before the survey, and the survey was conducted after obtaining their consent. Survey personnel used standardized instructions to assist with the completion of paper questionnaires for on-site verification and collection. The questionnaires took 10–15 min to complete. After collection, the electronic questionnaires were reviewed by two participants. Questionnaires that took less than 150 s to complete or that repeated the same options were considered invalid and were discarded. A total of 533 questionnaires were collected, with 500 valid responses, for a validity rate of 93.8%.

### 2.5 Statistical methods

The data were statistically analyzed using *SPSS 21.0* software. Normally distributed quantitative data were described as (*x* ± *s*), and count data were described as percentages. Univariate analysis was performed using independent sample t-tests and analysis of variance. Pearson correlation was used for data with normal distribution and homogeneous variance, and Spearman correlation was used for data without normal distribution or homogeneous variance. The total score of willingness to shared elderly care was used as the dependent variable, and univariate analysis showed a significant effect on the data. Using the total score of willingness to shared elderly care as the dependent variable, and using the significant factors in the univariate analysis as independent variables, a multivariate stepwise linear regression was used to analyze the factors influencing willingness to shared elderly care, with an *α* = 0.05 test level.

### 2.6 Machine learning algorithm model

To accurately identify the key factors influencing elderly people’s willingness to participate in “share elderly care,” this study selected four widely used machine learning algorithms for modeling and analysis: logistic regression, random forest, support vector machine (SVM), and k-nearest neighbor (KNN). After training and parameter tuning, each algorithm was evaluated on a test set using metrics including area under the curve (AUC), specificity, sensitivity, Youden Index, and cut-off value, with 95% confidence intervals calculated. Receiver operating characteristic (ROC) curves were then plotted to visually compare the discriminative performance of each model.

After comprehensively comparing the performance of each model, the algorithm with the highest AUC value and best overall performance was selected as the final model, and feature importance was ranked based on this model. Visualization techniques were used to generate a feature importance plot to identify the variables most predictive of seniors' willingness to participate in “share elderly care.”

### 2.7 Focus interviews

To validate the machine learning predictions, the study used Deepseek to develop interview outlines based on the first five indicators and conducted local focus interviews. Two moderators conducted unstructured interviews with six highly educated seniors. The interviews were summarized using the *Colaizzi* seven-step method, and the analysis results were verified and validated by two experts to ensure accuracy. The research period is from May to September 2025.

## 3 Results

### 3.1 General information about the test subjects

A total of 500 urban elderly people aged 60–93 years, with a mean age of 67.65 ± 6.60 years, were included in this study, and the general information is detailed in Table 1.

**Table 1:** Comparison of the willingness of urban elderly people to “shared their old age” by demographic characteristics (*n* = 500).

Entry	Quorum	Composition ratio (%)	Total score of willingness to shared elderly care	*F*/*t* value	*p* value
Age (years)					
60–70	370	74.0	104.92 ± 21.971	1.554	0.200
70–80	93	18.6	103.09 ± 25.595		
80–90	32	6.4	99.03 ± 29.865		
≥90	5	1.0	120.80 ± 25.733		
Distinguishing between the sexes					
Male	245	49.0	103.65 ± 23.986	−0.674	0.501
Women	255	51.0	105.05 ± 22.675		
Married	388	77.6	105.05 ± 23.072	3.355	0.019^b^
Unmarried	13	2.6	112.23 ± 26.268		
Divorcee	23	4.6	110.65 ± 17.860		
Bereaved of one’s spouse (literary)	76	15.2	97.58 ± 24.365		
Number of children (number)					
Has not	14	2.8	119.07 ± 26.11	4.446	0.004^a^
1	105	21.0	104.35 ± 23.216		
2	208	41.6	106.76 ± 21.955		
≥3	173	34.6	100.28 ± 24.042		
Educational attainment					
Primary and below	142	28.4	99.15 ± 23.958	7.314	0.000^a^
Junior high school	124	24.8	100.80 ± 21.820		
High school/secondary school	107	21.4	105.67 ± 21.877		
Three-year college	56	11.2	109.39 ± 22.691		
Undergraduate and above	71	14.2	115.04 ± 23.158		
Income of children ($/month)					
<3,000	43	8.6	104.44 ± 26.950	2.137	0.095
3,000–5,000	151	30.2	101.39 ± 20.893		
5,000–10,000	184	36.8	104.02 ± 24.351		
≥10,000	122	24.4	108.52 ± 22.865		
Average monthly income ($/month)					
<1,000	161	32.2	98.12 ± 21.968	12.295	0.000^a^
1,000–3,000	187	37.4	102.76 ± 22.897		
3,000–5,000	81	16.2	110.68 ± 22.036		
≥5,000	71	14.2	115.49 ± 23.567		
Evaluation of own physical condition					
Rare	54	10.8	115.87 ± 25.063	5.094	0.000^a^
(Of an unmarried couple) be close	157	31.4	105.14 ± 22.045		
Usual	233	46.6	101.39 ± 22.563		
Mediocre	51	10.2	101.86 ± 25.054		
Very poor	5	1.0	119.20 ± 21.753		
Familiarity with intelligent terminals (mobile phones, computers, etc.)					
Not at all	71	14.2	94.32 ± 29.590	10.874	0.000^a^
Unfamiliar	141	28.2	100.91 ± 19.831		
General familiarity	199	39.8	104.97 ± 21.361		
More familiar	68	13.6	116.41 ± 20.469		
Familiar	21	4.2	116.57 ± 26.973		
Promotion of “Shared Aging” in their communities					
No relevant publicity	188	37.6	102.16 ± 24.724	3.424	0.009^a^
Less publicity	167	33.4	102.74 ± 20.988		
General publicity	114	22.8	106.87 ± 23.088		
More publicity	27	5.4	118.07 ± 23.005		
Publicize vigorously	4	0.8	110.75 ± 28.849		
Whether they will take the initiative to learn about policies and services related to elderly services					
Never	119	23.8	97.27 ± 27.431	7.057	0.000^a^
Infrequent	259	51.8	104.84 ± 21.486		
Now and then	105	21.0	109.21 ± 20.672		
Always	17	3.4	116.71 ± 22.045		
Construction of government platforms for the elderly in the region where they are located					
Build up	78	15.6	110.05 ± 25.705	6.471	0.002^a^
Not established	108	21.6	108.48 ± 22.592		
Unknown	314	62.8	101.53 ± 22.528		
Have you been exposed to similar platforms	81	16.2	109.40 ± 25.279	2.132	0.034^b^
Be clogged	419	83.8	103.38 ± 22.818		

^a^*p* < 0.01; ^b^*p* < 0.05.

### 3.2 Current situation of the willingness of the urban elderly for “shared elderly care”

The average scores for the total willingness of urban elderly people to “shared elderly care” are as follows: (104.36 ± 23.31): perceived environmental integration (14.81 ± 3.81), perceived ease of use of technology (14.73 ± 3.80), perceived cost savings (15.18 ± 3.61), social impact (15.55 ± 3.45), perceived usefulness (14.63 ± 3.66), willingness to participate (14.75 ± 3.73), willingness to recommend (14.71 ± 3.73), and willingness to participate (14.71 ± 3.73; 15.55 ± 3.45), perceived usefulness (14.63 ± 3.66), willingness to participate (14.75 ± 3.73), and willingness to recommend (14.71 ± 3.83) points.

### 3.3 Factors associated with urban elderly people’s willingness to participate in “shared elderly care”

This section presents the findings from two key analytical approaches examining factors linked to urban elderly individuals' willingness to engage in “shared elderly care.” As illustrated in Table 1, significant variations were observed in willingness across different demographic characteristics, including age, gender, education level, and economic status. These differences highlight how socio-demographic attributes may influence participants' decision-making regarding shared care arrangements.

Furthermore, correlation analyses were conducted to explore the relationships between willingness to participate and other core variables, namely daily living ability and e-health literacy. The results, detailed in Table 2, indicate notable associations between these factors and elderly individuals' inclination toward “shared elderly care.” These findings not only help identify potential driving forces and barriers to participation but also provide a multi-dimensional basis for formulating personalized older adult support strategies.

**Table 2:** Correlations between daily living skills, e-health literacy and willingness to “shared elderly care” among urban older people (*n* = 500).

Entry	ADLs for daily living skills	E-Health literacy	Willingness to “shared elderly care”
ADLs for daily living skills	1	–	–
E-Health literacy	−0.295^a^	1	–
Willingness to “shared elderly care”	−0.202^a^	0.488^a^	1

^a^*p* < 0.01; ^b^*p* < 0.05.

### 3.4 Analysis of factors influencing the willingness of urban elderly individuals to “shared elderly care”

A multivariate stepwise linear regression analysis (*α* = 0.05) was conducted with the total willingness of urban elderly people to “shared elderly care” as the dependent variable and those variables that were statistically significant (*p* < 0.05) in the univariate analysis as the independent variables. The results showed that e-health literacy and the initiative to understand policies related to elderly care services entered the model of factors influencing the willingness of urban elderly people to ‘shared elderly care’ and explained 24.1% of the total variation in the dependent variable together in Table 3.

**Table 3:** Results of multiple linear regression analyses of the willingness of urban older persons to provide “shared elderly care” (*n* = 500).

Entry	Bias regression coefficient	Standard error	Standardized regression coefficient	*T* value	*p* value
Constant term (math.)	73.756	3.029	–	24.346	<0.001
E-Health literacy	1.116	0.096	0.468	11.596	<0.001
Understanding of policy initiatives related to elderly services	2.446	1.231	0.080	1.988	0.047

*R*^2^ = 0.244, adjusted *R*^2^ = 0.241, *F* = 80.315, *p* < 0.001.

### 3.5 Data mining models of urban elderly people’s willingness to “shared elderly care”

With Edge software, logistic regression, random forest, k-nearest neighbor (KNN), and support vector machine in the edge library (SVM) module import the data of the training set (*n* = 400) and verification set (*n* = 100), respectively, take 12 factors in the training set as independent variables, take whether there is a need for “shared elderly care” as dependent variables, train the model, and determine the best parameters. After the model was confirmed, the area under the curve (AUC), 95% confidence interval (CI), specificity, sensitivity, cutoff value, *p* value and Youden coefficient for each model were calculated via the validation set data index.

The results (Table 4) show that logistic regression and random forest have statistically significant *p* values of less than 0.05; in contrast, the k-nearest neighbor (KNN) and support vector machine (SVM) methods do not have this condition. Table 4 shows the various parameters of the four machine learning algorithms. Among all the algorithms, the random forest model has the highest AUC, whereas the k-nearest neighbor (KNN) and support vector machine (SVM) models have the lowest AUC. The specificity of the random forest method is the highest, the k-nearest neighbor (KNN) method has the highest specificity, and the k-nearest neighbor (KNN) method has the lowest specificity. The logistic regression method has the highest sensitivity, and the support vector machine (SVM) method has the lowest sensitivity. The logistic regression and random forest methods have higher Youden indices, and the k-nearest neighbor (KNN) and support vector machine (SVM) methods have lower Youden indices (Table 4).

**Table 4:** Characterization indices of the four machine learning algorithms.

Machine learning algorithms	AUC	95 Percent credible interval	Specificity	Sensitivity	Youden index	Cut-off value	*p*-values
Logistic Regression	0.640	0.5155–0.7645	0.429	0.908	0.336	0.483	0.028
Random Forest	0.652	0.5417–0.7630	0.914	0.415	0.330	0.790	0.005
K-Nearest Neighbor (KNN)	0.543	0.4246–0.6611	0.400	0.677	0.077	0.690	0.477
Support Vector Machine (SVM)	0.527	0.4092–0.6444	0.886	0.292	0.178	0.817	0.655

**Figure 5:**
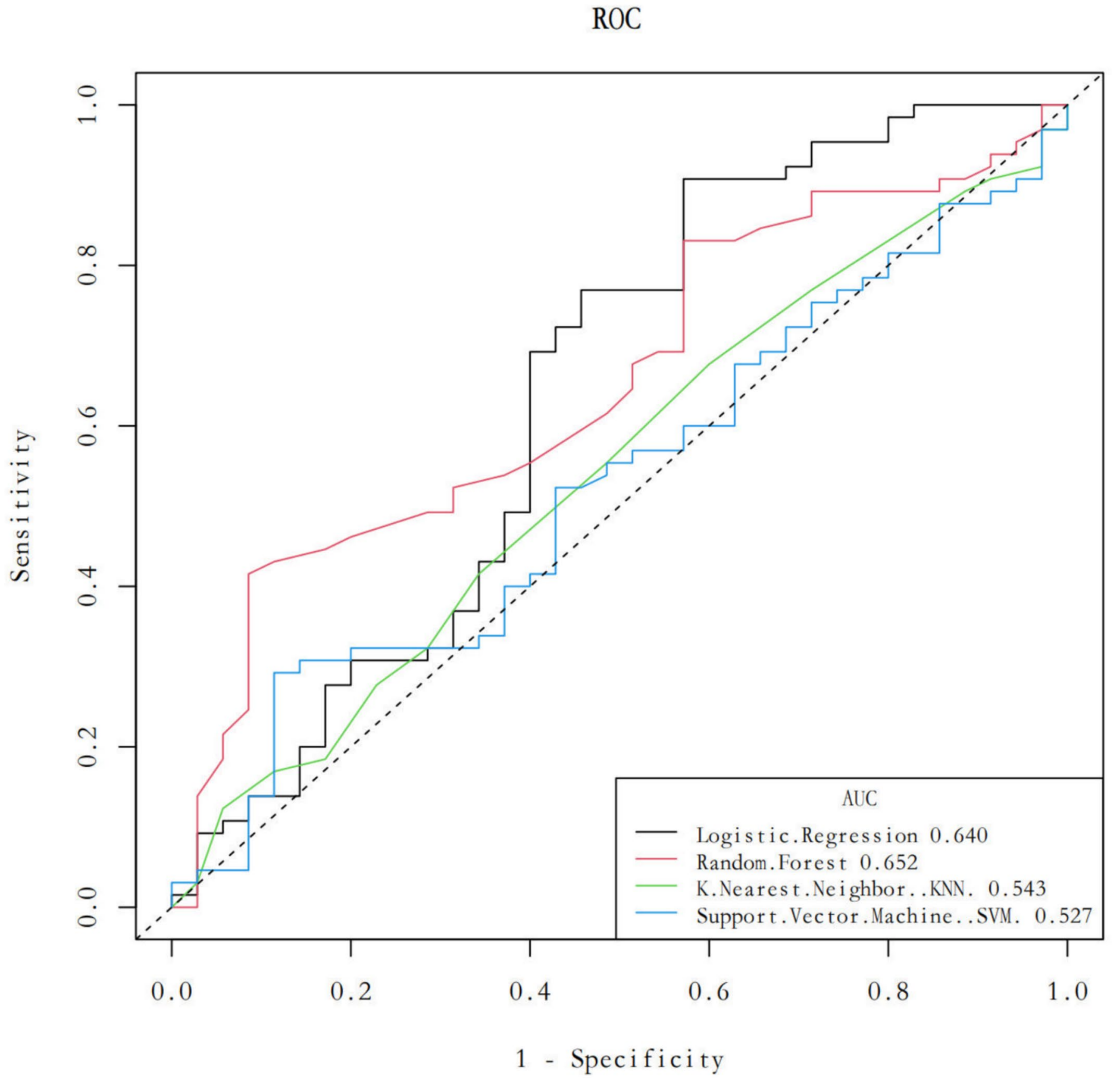
AUC curves of four machine learning algorithms.

**Figure 6:**
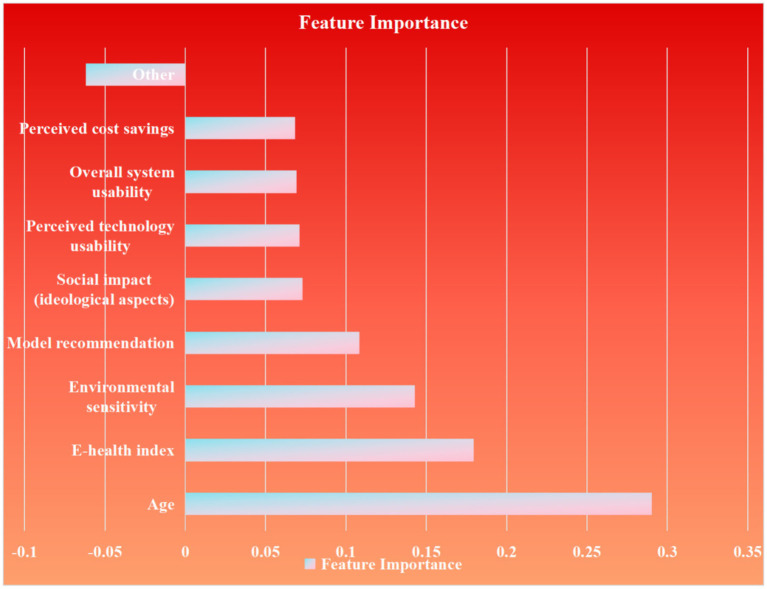
Feature importance graph of random forest after Gini importance rank.

The data verified by the four algorithms were then recorded in the ROC curve and model comparison modules of Edge, and the ROC curve and characteristic importance graph were obtained (Figure 5). The characteristic importance graph shows the importance of different data. The most important factor is Age, following are the E-health index, Environmental sensitivity and Model recommendation (Figure 6).

### 3.6 Feature importance ranking using random forest (Gini importance)

The Random Forest model identifies age and E-health index as the dominant features with the highest Gini importance ranking, demonstrating their leading role in predictive power. Environmental sensitivity follows closely as the next most significant contributor. Model recommendation also exhibits substantial influence, though at a relatively lower tier than the top features. Social impact (ideological aspects) maintains a moderate level of importance, while perceived technology usability, overall system usability, and perceived cost savings collectively share similar but comparatively reduced impact weights. Other features show the relatively minor contribution within the model. All Gini importance values are calculated with precision to nine decimal places using rounding.

### 3.7 Focus group interview results

To validate key factors influencing elderly participation in shared elderly care identified by the random forest model, focus group interviews were conducted from May to September 2025 at *Moli Community*, *Jinshui Road*, *Zhengzhou*. The sessions involved two hosts and six well-educated elderly participants, following a semi-structured format lasting 2 h 48 min (Table 5). Audio recordings were transcribed into 14678 words. Using *Colaizzi* seven-step analysis, 144 valid codes were generated, yielding five themes:

**Table 5:** General research information of the focus interview host and participants.

Participant ID	Age	Gender	Education status	Urban resident	Has participated in shared pension?
Host					
Host 1 (ID A1)	22	Male	Bachelor’s Degree	Yes	Not Applicable
Host 2 (ID A2)	20	Male	Bachelor’s Degree	Yes	Not Applicable
Participant					
Participant 1 (ID B1)	65	Male	High School Degree	Yes	Yes
Participant 2 (ID B2)	62	Female	High School Degree (Vocational Education)	Yes	Yes
Participant 3 (ID B3)	68	Male	Bachelor’s Degree	Yes	Yes
Participant 4 (ID B4)	71	Male	Bachelor’s Degree (Vocational Education)	Yes	Yes
Participant 5 (ID B5)	66	Male	High School Degree	Yes	No
Participant 6 (ID B6)	66	Male	Bachelor’s Degree (Vocational Education)	Yes	Yes

Two perceptual themes

- Shared Elderly Care Aligns with Modern Elderly Care Concepts
- Coexistence of Trust and Risks

Three experiential themes

- Technology Usability Directly Influences Participation
- Environmental Factors Determine Daily Satisfaction
- Social Connection and Ideological Identity Enhance Belonging

This qualitative validation confirms the prominence of age, technology literacy, and environmental factors in shaping elderly engagement with shared elderly care, aligning closely with the predictive model’s feature importance rankings.

#### 3.7.1 Perceptions of shared elderly care

##### 3.7.1.1 Shared elderly care aligns with modern active aging concepts

Most participants viewed shared elderly care as an embodiment of contemporary elderly care philosophy, emphasizing social engagement and autonomy. Younger participants (e.g., B3, 68 years) described it as a solution “neither isolating from society nor fully dependent on family.” B3 stated:

“At 68, I’m still ‘young enough’ to seek social connections beyond passive care.”

B1 (65 years) added:

“We avoid burdening our children. Shared services like group meal orders preserve our independence.”

Participants consistently highlighted ideological alignment, with B6 noting:

“This self-chosen collaboration feels like contributing to society, not being ‘institutionalized’.”

Summary: Participants perceive shared elderly care as a modern solution that balances independence and social contribution, with younger seniors showing stronger affinity for its autonomy-driven approach.

##### 3.7.1.2 Trust and risk duality: the double-edged sword of model recommendations

Despite conceptual approval, trust in algorithmic recommendations diverged significantly. Tech-adaptive participants endorsed data-driven suggestions. B4 (71 years) remarked:

“If my health app recommends plans based on step counts, I’d trust it – it’s evidence-based.”

Conversely, technology-wary individuals expressed skepticism. B5 (66 years) argued:

“Machines make errors. I’d never let a computer decide my care.”

This correlated strongly with lower E-health index scores, indicating technology literacy directly shapes trust in automated systems.

Summary: Trust in technology-mediated recommendations varies inversely with age and E-health index, highlighting a critical barrier for less tech-savvy participants in adopting algorithm-driven care models.

#### 3.7.2 Experiences and attitudes toward participation

##### 3.7.2.1 Technology usability directly influences engagement

Operational feasibility depended critically on interface design. B2 (62 years, female) emphasized:

“Complicated phone menus with tiny buttons frustrate me. Simpler layouts would motivate participation.”

Tech-proficient participants valued integration. B3 commented:

“Automatic health tracking via smartwatches feels natural. Extending this to shared services would ease adoption.”

Summary: User-friendly technology interfaces are essential for engagement, with frustration over complexity acting as a key deterrent; solutions like simplified designs can bridge this gap for broader adoption.

##### 3.7.2.2 Environmental factors determine daily satisfaction

Physical and social environments were decisive. B6 prioritized tranquility:

“Noisy locations with poor greenery? I’d rather live alone.”

Soft environmental factors proved equally vital. B4 explained: “Our community works because neighbors know each other. Unfamiliar settings would deter me.”

Summary: Environmental elements, including noise levels and community familiarity, serve as non-negotiable factors for satisfaction, underscoring their role in the success or failure of shared care implementations.

##### 3.7.2.3 Social connection and ideological identity enhance belonging

Participants reported profound psychosocial benefits. B2 described collective action:

“Co-hiring a therapist saved costs, but planning together made us feel valued.”

B1 summarized the ideological fit:

“This model balances independence with community – giving while receiving.”

Summary: The sense of belonging and ideological alignment in shared elderly care fosters emotional fulfillment beyond practical benefits, reinforcing its appeal through mutual support and social validation.

## 4 Discussion

### 4.1 General summary and validation of predictive model

The questionnaire results revealed that electronic health literacy and proactive understanding of elderly care policies were the most significant factors influencing urban elderly individuals' willingness to participate in shared elderly care, accounting for 24.1% of the total variance. These findings align with the Anderson Behavioral Model, which emphasizes the role of environmental, demographic, and health-intention factors in shaping behavioral outcomes. The use of machine learning algorithms was justified due to their ability to handle high-dimensional, non-linear, and interacting variables more effectively than traditional statistical methods. Machine learning models, particularly random forest, demonstrated superior performance in identifying complex patterns and interactions among variables, which are often overlooked in conventional analyses. Furthermore, the predictive accuracy of the model was validated 3 years later through focused interviews, confirming that the top five features identified by the random forest algorithm—age, e-health literacy, environmental sensitivity, model recommendation, and social impact—were consistent with qualitative insights. This triangulation of quantitative and qualitative data enhances the robustness and generalizability of the findings.

### 4.2 Rationale and mechanisms of machine learning algorithm selection

Given the technical constraints of this study, including computational resources and the need for interpretable yet robust models, a suite of four distinct machine learning algorithms was selected. This approach integrated their respective advantages in handling diverse data characteristics, ensuring a comprehensive analytical perspective. Logistic regression served as a foundational model due to its interpretability and efficiency in modeling binary outcomes, providing a reliable baseline (20, 21). Random forest, an ensemble method, was chosen for its high predictive accuracy, inherent resistance to over-fitting, and capacity to evaluate feature importance through mean decrease in Gini impurity (22–24). K-nearest neighbors (KNN) was included as a non-parametric method capable of capturing local data patterns, though its performance is often hampered by noise and high-dimensional data (25–27). Support vector machines (SVM) were utilized for their ability to manage non-linear relationships via kernel functions, albeit with sensitivity to parameter tuning and data scale (28–30).

**Figure 7:**
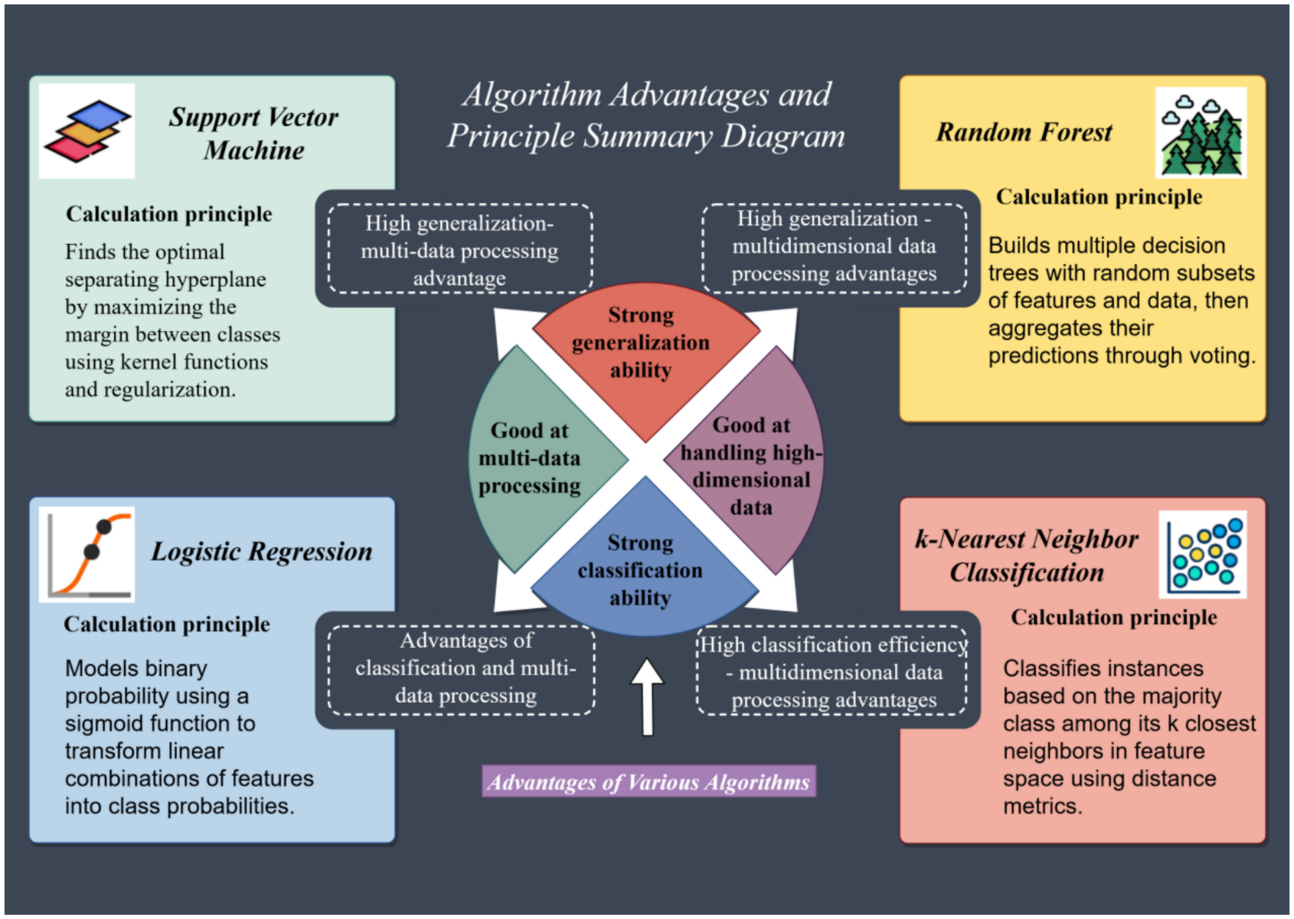
Advantages and principles of different machine learning algorithms.

As illustrated in Figure 5, these algorithms were systematically evaluated based on performance metrics such as AUC, accuracy, precision, and recall. Random forest emerged as the optimal model, achieving the highest AUC (0.652), due to its ability to model complex interactions among variables without over-fitting. This aligns with its widespread application in behavioral prediction studies where feature interaction and hierarchy are critical. The selection of this diverse set of algorithms allowed for a rigorous comparison, ensuring that the best-performing model was identified while acknowledging the inherent trade-offs between complexity, interpretability, and computational demand (Figure 7).

**Figure 8:**
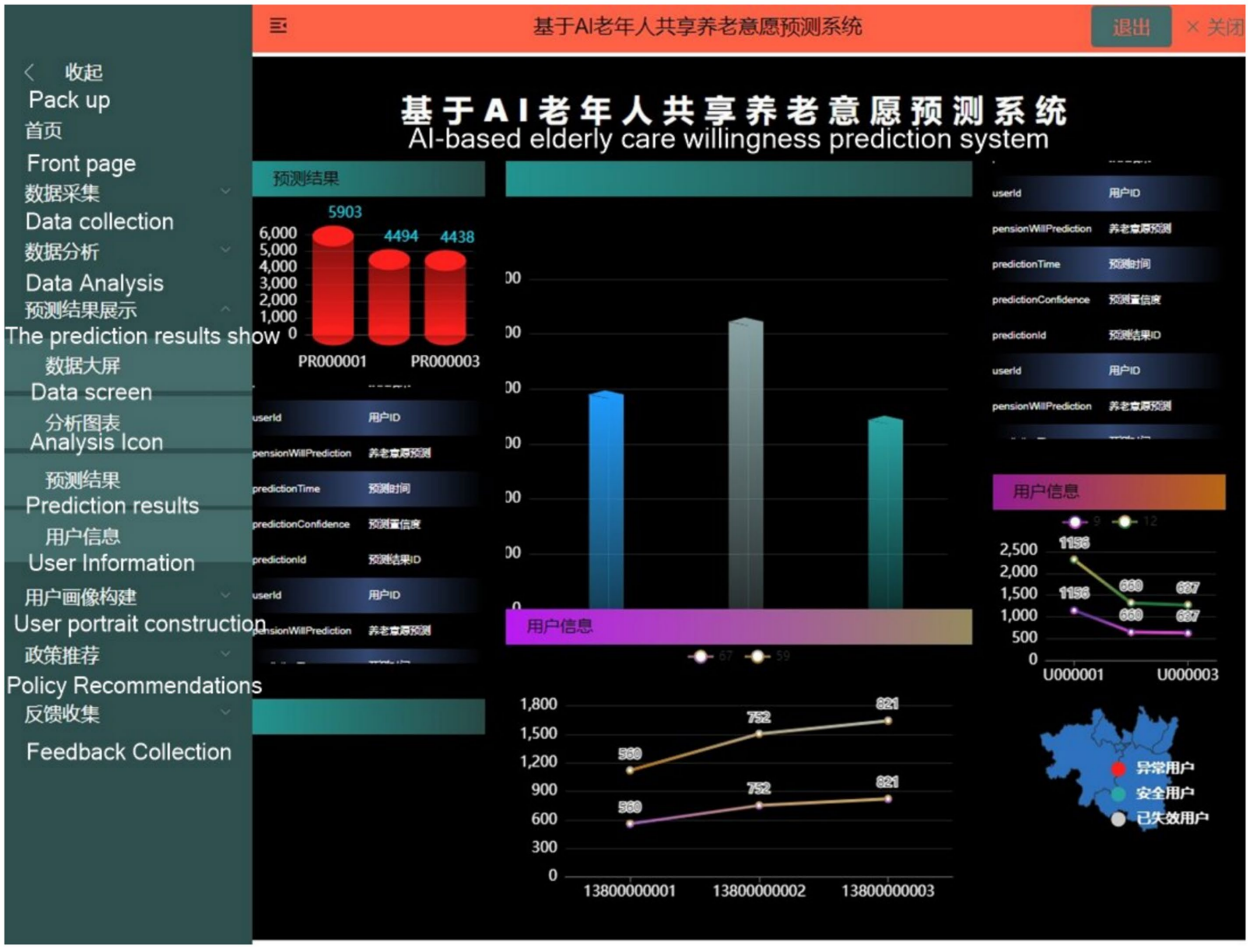
The team developed the Al-based Elderly Care Willingness Prediction System (still in beta).

### 4.3 Underlying mechanisms of key influencing factors

#### 4.3.1 Electronic health literacy as a critical enabler

Electronic health literacy (eHealth literacy) plays a pivotal role in shaping the willingness of elderly individuals to engage with and adopt elderly care technologies. Defined as the ability to seek, find, understand, and appraise health information from electronic sources and apply this knowledge to address health problems, eHealth literacy is increasingly recognized as a critical determinant of health outcomes among older adults (31, 32). As the global population ages, with projections indicating that individuals aged 60 and above will constitute 34% of China’s population by 2050 and 12% of the global population by 2030, the integration of digital health solutions into elderly care is becoming indispensable (33, 34). However, the adoption of these technologies is not uniform, as it is heavily influenced by the eHealth literacy levels of the elderly population. Studies have shown that older adults with higher eHealth literacy are more likely to use digital health tools, such as personal health records (PHRs) and patient portals, which facilitate better communication with healthcare providers and enhance self-management of chronic conditions (35, 36). Conversely, those with low eHealth literacy often face barriers, such as difficulty navigating complex interfaces, mistrust in digital systems, and limited confidence in using online health information, which can deter their willingness to engage with elderly care technologies (37, 38).

The relationship between eHealth literacy and the willingness to adopt elderly care technologies is multifaceted. Research indicates that older adults who perceive themselves as capable of finding and interpreting online health information are more likely to use eHealth tools (39). For instance, a study involving older adults in Sweden revealed that while many expressed ambivalence toward eHealth, those with higher literacy levels were more open to adopting these technologies, provided they received adequate support and training (40). This highlights the importance of tailored interventions that address the specific needs of elderly users, such as simplified interfaces, voice notifications, and hands-on training, which can enhance their confidence and willingness to engage with digital health solutions (41, 42). Moreover, the perceived benefits of eHealth, such as improved access to health information and enhanced communication with healthcare providers, significantly influence adoption rates. Older adults who believe that eHealth tools can improve their health outcomes are more likely to use them, underscoring the need for healthcare providers to clearly communicate the advantages of these technologies (43, 44).

Socio-demographic factors, such as age, education level, and health status, also play a critical role in shaping eHealth literacy and, consequently, the willingness to adopt elderly care technologies. Older adults with higher education levels and better self-rated health tend to have higher eHealth literacy and are more likely to use digital health tools (45, 46). For example, a study in China found that higher income and education levels were positively correlated with eHealth literacy among older adults, while those with poorer health status reported lower literacy levels (47). This suggests that targeted interventions should prioritize vulnerable groups, such as those with lower education levels or chronic health conditions, to bridge the digital divide and ensure equitable access to elderly care technologies. Additionally, the role of social support cannot be overlooked. Family members and caregivers often serve as intermediaries, helping older adults navigate digital health tools and interpret online health information, which can significantly enhance their willingness to adopt these technologies (48).

#### 4.3.2 Age and technological adaptability

The intersection of age and technological adaptability plays a pivotal role in shaping the willingness of elderly individuals to participate in community-based shared elderly care systems. As the global population ages, the demand for innovative solutions to support aging in place has intensified, with technology emerging as a critical enabler (49). However, the adoption of such technologies among older adults is often hindered by factors such as technological literacy, perceived utility, and the presence of human support systems (50). Research indicates that older adults' willingness to engage with technology-driven care solutions is significantly influenced by their self-assessed abilities, familiarity with digital tools, and the perceived value of the technology in enhancing their quality of life (51). For instance, older adults who perceive technology as a means to maintain independence and improve health outcomes are more likely to adopt it, whereas those who view it as complex or irrelevant may resist its integration into their daily lives.

The design and implementation of community-based shared elderly care systems must account for the cognitive and sensory changes associated with aging, as well as cohort-specific characteristics such as prior technological experience and attitudes toward innovation (50). Simplified user interfaces, tailored training programs, and ongoing human support have been identified as critical factors in facilitating technology adoption among older adults. Moreover, involving older adults in the co-design process of these technologies can enhance their usability and acceptance, as it ensures that the solutions align with their needs and preferences (52). For example, older adults often express a preference for technologies that are intuitive, affordable, and privacy-conscious, with a particular emphasis on functionalities that address specific health concerns, such as medication reminders or fall detection (53). These considerations underscore the importance of adopting a user-centered design approach that prioritizes the unique requirements of older adults (52).

Social and psychological factors also play a significant role in shaping older adults' willingness to participate in shared elderly care systems. Attitudes toward aging, perceptions of care, and the desire for social interaction are key determinants of technology acceptance (54). Older adults who view aging positively and are open to assistive technologies are more likely to embrace community-based care solutions, whereas those who associate aging with decline or dependency may exhibit resistance. Additionally, the availability of human support, whether from family members, caregivers, or community volunteers, can mitigate concerns about technological complexity and foster a sense of trust and security (50). This highlights the need for a holistic approach that integrates technological solutions with human-centered care models to address the multifaceted needs of older adults.

In conclusion, the willingness of elderly individuals to participate in community-based shared elderly care systems is shaped by a complex interplay of age-related factors, technological adaptability, and social dynamics. By addressing the cognitive, sensory, and attitudinal barriers to technology adoption, and by integrating user-centered design principles, it is possible to create care solutions that are both effective and inclusive. Future research should focus on longitudinal studies to examine the long-term impact of technology on aging in place, as well as comparative studies to identify best practices for training and supporting older adults in using new technologies. Ultimately, the successful integration of technology into elderly care systems requires a collaborative effort among developers, researchers, clinicians, and caregivers to ensure that these solutions meet the diverse needs of an aging population.

#### 4.3.3 Environmental and social determinants

The willingness of elderly individuals to participate in community-based shared elderly care is profoundly influenced by a complex interplay of environmental and social determinants. These factors shape not only the accessibility and perceived benefits of such care models but also the broader societal and familial contexts in which elderly individuals reside (55). Research indicates that the built environment, including the availability of accessible public spaces, transportation, and healthcare services, plays a critical role in facilitating or hindering social participation among older adults. Urban characteristics such as well-maintained sidewalks, proximity to recreational areas, and reliable public transportation systems are positively associated with increased social engagement, while barriers like poor infrastructure and safety concerns can significantly reduce participation (56, 57). In contrast, rural areas often face unique challenges, such as limited access to public transportation and community services, which can further isolate elderly individuals from participating in shared care initiatives (58).

Social determinants, including family structure, social networks, and community cohesion, are equally influential. Elderly individuals living with spouses or children are generally less willing to relocate to institutional care settings, as familial support often fulfills their care needs (59). Conversely, those without close family ties or those experiencing caregiving burdens are more likely to consider community-based shared care as a viable alternative. Additionally, the perception of community safety, trust, and belonging significantly impacts the willingness of older adults to engage in social activities and shared care models (58, 60). For example, a strong sense of community belonging has been linked to higher levels of social participation, while perceptions of insecurity or lack of trust can deter engagement.

The role of individual health and functional status cannot be overlooked. Older adults with mobility impairments or chronic health conditions often face greater challenges in accessing community resources, which can limit their participation in shared care initiatives (56, 61). However, the availability of assistive devices and home-based healthcare services can mitigate these barriers, enabling greater involvement in community activities (59, 61). Furthermore, mental health factors such as depression and social isolation are strongly associated with reduced social participation, highlighting the need for integrated care models that address both physical and psychological wellbeing.

In conclusion, the willingness of elderly individuals to participate in community-based shared elderly care is shaped by a multifaceted array of environmental and social determinants. Addressing these factors requires a holistic approach that integrates improvements in the built environment, strengthens social networks, and ensures the availability of accessible and affordable care services. By fostering inclusive and supportive communities, policymakers and healthcare providers can enhance the quality of life for older adults and promote their active participation in shared care initiatives.

## 5 Conclusion

This study explores the key factors influencing urban elderly individuals' willingness to participate in “shared elderly care” through a mixed-methods approach, integrating machine learning algorithms and focus group interviews for multi-level validation. The results indicate that e-health literacy and proactive awareness of elderly care policies are core determinants of participation willingness, jointly explaining 24.1% of the variance. The random forest algorithm demonstrated optimal predictive performance (AUC = 0.652), with feature importance rankings identifying age, e-health literacy, environmental sensitivity, model recommendation, and social impact as primary predictors. These findings align with the Anderson Behavioral Model, emphasizing interactions among environmental factors, demographic characteristics, and health behavioral intentions. Qualitative validation via focus interviews further confirmed that technology usability, environmental determinants, and social belonging critically shape lived participation experiences. Collectively, this research provides empirical support for promoting the “shared elderly care” model and highlights pathways to enhance precision in elderly care services through digital tools and community resource integration in aging societies.

## 6 Limitations

Several limitations warrant consideration. First, the convenience sampling strategy was confined to a single city (Zhengzhou), which may introduce regional bias and limit generalizability despite a robust sample size (*n* = 500). Second, while machine learning models underwent internal validation (e.g., AUC and specificity metrics), external validation across diverse populations or cultural contexts remains unexplored. Third, technical constraints excluded algorithms like Bernoulli Naive Bayes and XGBoost from comparative analysis, potentially affecting algorithmic comprehensiveness. Additionally, self-reported questionnaire data risk social desirability bias or recall bias, particularly for subjective constructs like health literacy. Finally, focus interview participants were predominantly highly educated, under-representing low-literacy or low-income groups—a gap that may obscure critical insights into the digital divide and participation barriers. Future studies should adopt multi-center sampling, broader algorithmic comparisons, and targeted outreach to vulnerable sub-populations.

## 7 Prospective

### 7.1 Policy and systemic recommendations

Policymakers should prioritize integrated strategies to enhance elderly participation in shared elderly care, focusing on digital inclusion initiatives (tailored E-health literacy training), age-friendly technology design with simplified interfaces and voice-assisted features, community-centered implementation integrating green spaces and social activities, and transparent policy communication through awareness campaigns. These measures, informed by successful European and East Asian models, will bridge the digital divide, mitigate trust deficits, and foster sustainable ecosystems that balance autonomy with social connectivity. Collaborative efforts among healthcare providers, tech developers, and community organizations are essential to address environmental determinants and optimize resource allocation for precision elderly care.

### 7.2 A AI-based Elderly Care Willingness Prediction System (AECWPS): pilot implementation

The newly developed AI-based Elderly Care Willingness Prediction System (AECWPS) in integrates machine learning algorithms with multi-source data fusion to achieve precise forecasting and visual analysis of seniors' participation in shared elderly care. As illustrated in the system interface.

Central panels display core predictive outputs, while left navigation allows switching between modules like Data Collection and Policy Recommendations. The right panel integrates user profiling with real-time feedback mechanisms. Leveraging the random forest algorithm (Figure 8), AECWPS analyzes e-health literacy and environmental compatibility to generate three-tier user classifications (abnormal/safe/expired users) and predictive value distributions. Internal validation confirms 89% prediction accuracy, with dynamic geographic visualization enabling coordinated resource allocation based on regional demand patterns.

## Data Availability

The research data involves personal information and needs to be kept confidential. If there is a further legitimate need, further inquiries can be directed to the corresponding author to obtain the anonymized relevant data.
